# *Duhaldea pterocaula* (Franch.) Anderb. Attenuates Nociception and Inflammation *via* GABA_A_ Receptors

**DOI:** 10.3389/fphar.2021.753128

**Published:** 2021-11-02

**Authors:** Chunli Huang, Changsheng Dong, Yanan Zhu, Yang Yu, Huizi Jin, Yan Zhang

**Affiliations:** ^1^ School of Pharmacy, Shanghai Jiao Tong University, Shanghai, China; ^2^ Cancer Institute of Traditional Chinese Medicine, Longhua Hospital, Shanghai University of Traditional Chinese Medicine, Shanghai, China

**Keywords:** *Inula pterocaula* Franch, GABA, analgesic, anti-inflammatory effect, GABA_A_ receptors

## Abstract

*Duhaldea pterocaula* (Franch.) Anderb, also known as *Inula pterocaula* Franch (*I. pterocaula*), is a folk medicine of the Yi nationality in China. The Inula plants display various biological activities, including anti-nociceptive and anti-inflammatory properties. *I. pterocaula* has been traditionally used for the treatment of bronchitis, vasculitis, and dizziness. However, very few studies have been reported on the pharmacology of *I. pterocaula*. The present study aims to characterize the anti-nociceptive and anti-inflammatory properties of *I. pterocaula* and explore the underlying mechanism. *I. pterocaula* was extracted by 95% ethanol and further portioned with petroleum ether, ethyl acetate (EA) and n-butanol, sequentially, to obtain corresponding factions with different polarities. The EA fraction (IPEA) was found to be one of the most effective fractions. It demonstrated potent analgesic effects in both acute and inflammatory pain mouse models, and caused no anti-nociceptive tolerance. Furthermore, IPEA improved the tolerance of mice to morphine. IPEA also showed potent anti-inflammatory effects on LPS-induced septic mice. BIC, a GABA_A_R antagonist, reversed the effects of IPEA in pain and inflammation models. Collectively, GABA_A_Rs play a key role in the pharmacological effects of IPEA. *I. pterocaula* may be useful as a complementary or alternative therapeutic agent for the treatment of pain and inflammation.

## Introduction

Pain is a complex biological response and usually accompanies inflammation and injury. Acute pain is often evoked by acute inflammation and plays an important role to protect the injured tissue. Chronic pain is maladaptive and associated with inflammation (Oh et al., 2001; Basbaum et al., 2009; Dawes et al., 2011; Ji et al., 2018). Inflammatory mediators, such as nitric oxide and pro-inflammatory cytokines (eg., TNF-α, interleukin 1β), are able to activate or sensitize nociceptors and thus elicit chronic pain (Oh et al., 2001; Basbaum et al., 2009; Gold and Gebhart, 2010). Pain management is currently limited, especially in chronic pain (Loeser and Melzack, 1999). The opiate drugs and nonsterioidal anti-inflammatory drugs (NSAIDs) are common and effective against moderate to severe pain (Bowsher, 1993; Derry et al., 2015). However, these drugs cause severe side effects, including analgesia tolerance (Dodrill et al., 2011). Therefore, there is an urgent need for new analgesic alternatives.

GABA_A_ receptors (GABA_A_Rs) are Cl-selective ligand-gated ion channels which are formed from 19 subunits (Olsen, 2018; Zhu et al., 2018). GABA_A_Rs are the natural target of gamma-aminobutyric acid (GABA), a main inhibitory neurotransmitter of the mammalian central nervous system (Farrant and Nusser, 2005; Zhu et al., 2018). In general, GABA is produced by l-glutamic acid under the catalysis of glutamate decarboxylase (GAD). Synthesized GABA is stored in synaptic vesicles and released into the synaptic cleft upon nerve impulse to act on its two distinct classes of receptors, including GABA_A_Rs and GABA_B_Rs. GABA binds to GABA_A_Rs at the α-β interface, enabling an influx of chloride ions through the central pore, causing receptor hyperpolarization, thus inhibiting neurotransmission (Lüddens and Korpi, 1995; Zhu et al., 2018). The genetic approach and pharmacological intervention have been employed to study the GABA/GABA_A_Rs neurotransmission system (Fischer, 2017; Ge et al., 2019; Neumann et al., 2021). Many drugs target GABA_A_Rs, thus affect GABAergic functions. These drugs are of clinical importance, and are widely used in the treatment of anxiety disorder, epilepsy, insomnia, spasticity, and aggressive behavior (Rudolph and Knoflach, 2011). Although there are no significant clinical analgesic and anti-inflammatory drugs with a GABAergic mechanism of action, recent studies showed that central and periphery GABA_A_Rs play a major role in nociception and inflammation. Considerable evidence supported that facilitating GABAergic inhibition was able to reverse pathological pain states in mice and also pointed to the associations between GABA_A_R and inflammatory disorders in brain and peripheral organs; GABA was able to decrease the pro-inflammatory mediator production and ameliorate inflammatory symptom (Knabl et al., 2008; Munro et al., 2009; Bhat et al., 2010; Seifi et al., 2018; Neumann et al., 2021).

*Duhaldea pterocaula* (Franch.) Anderb., also known as *Inula pterocaula Franch* (*I. pterocaula*), belongs to Inula L. genus, Asteraceae. It is mainly distributed in southern and western Sichuan Province, as well as in the northern and northwestern Yunnan Province in China. *I. pterocauusla* is a folk medicine of the Yi nationality. The fresh or dried whole plant of this species is routinely used in traditional medicine. Approximately 100 species of Inula plants across Asia, Europe, and Africa, exhibit diverse biological activities, such as anti-nociception and anti-inflammation (Amin et al., 2013). Some of the documented traditional uses of *I. pterocaula* include bronchitis, vasculitis and dizziness treatment (Committee, 1979; Jia and Li, 2005). However, scientific pharmacological studies on *I. pterocaula* remain under-exploited. Our previous study has identified and characterized some anti-inflammatory components from *I. pterocaula* (Zhu et al., 2019). We’ve also evaluated the *in vitro* anti-inflammatory effects of *I. pterocaula* extracts of different polarities, and found that the ethyl acetate extract of *I. pterocaula* (IPEA) was the most effective fraction (Huang et al., 2021).

Therefore, these clues drew our interest and promoted us to explore the potential anti-nociceptive effect of *I. pterocaula.* In our ongoing projects to search novel analgesics from natural products, we’ve shown interest in different pharmacological targets related to pain modulation (Long et al., 2014; Zhang et al., 2014; Zhang et al., 2014; Wang et al., 2016). *I. pterocaula* was one of the natural products that we screened to target GABAergic transmission.

## Materials and Methods

### Materials

Parthenolide (PAR), complete Freund’s adjuvant (CFA), lipopolysaccharides (LPS) and naloxone were purchased from Sigma-Aldrich (St. Louis, MO) (+)-Bicuculline (BIC) was purchased from Topscience (Shanghai, China). 3-mercaptoethanol (3-MP) was purchased from Meryer (Shanghai, China). Morphine was obtained from Northeast Pharmaceutical Group (Shenyang, China). NO detection kit was obtained from Beyotime Institute of Biotechnology (Jiangsu, China). The ReverTra Ace qPCR RT kit was purchased from TOYOBO Co., Ltd (Osaka, Japan). IL-6 ELISA kit and IL-1β ELISA kit were obtained from Thermofisher Co., Ltd (MA, United States) and TNF-α ELISA kit was purchased from Neobioscience (Shenzhen, China). Antibodies were purchased as following: Anti-ERK, JNK, p38, iNOS, p65, AKT, and phosphor-ERK, -JNK, -p38, -p65, -AKT were purchased from Cell Signaling Technology (Danvers, MA, United States). Anti-β-actin was purchased from Bioss (Beijing, China). Anti-GABA_A_ Receptor alpha 2 (anti-GABA_A_Rα2) was purchased from Abcam (Cambridge, United Kingdom). GAPDH was obtained from Proteintech (Wuhan, China). IRDye®800CW secondary antibodies were purchased from LI-COR (Lincoln, NE, United States).

### Plant Extraction

The plant of *I. pterocaula* was collected from Kunming, Yunnan Province of China and authenticated by Kunming Zhifen Biotechnology Co., Ltd. A voucher specimen (No. 20181YJYEJ) was deposited at School of Pharmacy, Shanghai Jiao Tong University. *I. pterocaula* was extracted as previously described (Zhu et al., 2019). In brief, the dried whole plants of *I. pterocaula* (11 kg) were cut into small pieces and reflux extracted with 95% ethanol (each time 80 L, three times) and then evaporated under negative pressure to give crude extract (1,335 g). The extract was further partitioned with petroleum ether (PE), ethyl acetate (EA) and n-butanol (n-BuOH) (each time 3 L, three times) to obtain three fractions, including PE fraction (382 g), EA fraction (220 g, IPEA) and n-BuOH fraction (246 g), respectively. An aliquot of IPEA fraction was further used in this pharmacological study. IPEA was dissolved in the vehicle which was the mixture of hydrogenated castor oil, ethyl alcohol and saline (10:5:85).

### Cell Culture

The mouse macrophage cell line (RAW 264.7) was purchased from China Cell Line Bank (Beijing, China). The cells were grown in RPMI-1640 supplemented with 10% heat-inactivated FBS at 37°C under a humidified atmosphere of 5% CO_2_.

### Animals

Male C57BL/6 mice (6–8 weeks old) were purchased from Shanghai Lingchang Biological Technology Co., Ltd. (Shanghai, China) and GemPharmatech (Jiangsu, China). The animals were housed in the Laboratory Animal Center of Shanghai Jiao Tong University (SJTU, Shanghai, China), at controlled room temperature of 22–24°C and a relative humidity of 55 ± 10% with a 12 h light/dark cycle. The animals were acclimatized for at least 1 week prior to the experiments. The animals are free access to water and food. The animal studies were performed in accordance with the Guidelines for Care and Use of Laboratory Animals of SJTU. The animal facilities and protocols were approved by the Animal Ethic Committee of SJTU (approved number “A2017071”). The performers who conducted the behavioral testing were blinded to drug administration groups.

### Bioassay for NO Production

The accumulation of nitrite was calculated by the production of nitric oxide (NO), which was measured by Griess assay as our previous work (Wu et al., 2017). In brief, RAW 264.7 macrophage cells were seeded in a 96-well plate at a density of 3×10^4^ cells/well and pretreated with IPEA (10 μg/ml) for 1 h before LPS stimulation (100 ng/ml). After 24 h, an equal volume (50 μl) of the supernatant was mixed with Griess reagent on a 96-well plate. The mixture was allowed to react for 10 min, and the release of NO was measured at 540 nm on a microplate reader.

### Quantitative Real-Time PCR

Real-Time PCR (RT-PCR) was performed to quantify the transcription of IL-6, IL-1β, TNF-α, GABA_A_Rα2 and Gad 2. The following primers were used:

The primers for the mouse reference gene GAPDH (5′- AGG​TCG​GTG​TGA​ACG​GAT​TTG-3’; 3′- TGT​AGA​CCA​TGT​AGT​TGA​GGT​CA-5′). The Quantitative RT-PCR was operated according to the following conditions: 94°C for 3 min, 40 cycles of denaturation at 95°C for 10 s, annealing at 58°C for 30 s, and extension at 72°C for 20 s, followed by a final extension at 72°C for 10 min. The quantified fold changes in messenger RNA (mRNA) in each sample were normalized to GAPDH expression and calculated using the 2exp (−ΔΔCt) method.

### Measurements of IL-6, IL-1β and TNF-α by ELISA

RAW 264.7 macrophages were seeded in a 24-well plate at a density of 1.0 × 10^5^ cells/well. After 14 h, cells were treated with IPEA for 1 h and followed by LPS stimulation (100 ng/ml). After 4 h, the supernatants of cultured medium were collected. Liver tissue samples of mice were processed with a tissue homogenizer. The supernatant was obtained by centrifuging at 4 °C, 4,000 rpm for 20 min. Blood samples were collected by centrifuging at 4°C, 4,000 rpm for 10 min. IL-6, IL-1β, and TNF-α in the supernatants were determined by ELISA kits according to the manufacturer’s instructions.

### Western Blot

Cells were seeded into a 12-well plate at a density of 2.0×10^5^ cells/well. After 14 h, cells were pretreated with IPEA for 1 h, and then stimulated with LPS (100 ng/ml) for 24 h for iNOS, or 30 min for ERK and P38, respectively. The mice spinal cord samples were treated with tissue homogenizer and quantified by BCA Protein Assay Kit. An equal amount of protein was prepared by SDS-PAGE sample loading buffer and heated at 99°C for 10 min and separated on 10% SDS-PAGE and transferred onto a nitrocellulose membrane (Whatman Protran, Dassel, Germany). At room temperature, the membrane was blocked with 5% non-fat milk for 1 h, and then incubated with specific primary antibody overnight at 4°C. The next day, the membrane was washed three times with TBST, and then incubated with IRDye®800CW secondary antibody. The 800 nm channel on Odyssey® CLX Infrared Imaging System was used to scan the membrane. The NIH ImageJ software was used to quantify the immunoreactive bands.

### Mouse Studies

#### Hot Plate Assay

Hot plate assay was conducted to study the central anti-nociceptive effect of IPEA in mice using hot plate analgesia meter. The method was originally described by Woolfe et al. (Woolfe and Macdonald, 1994). Briefly, mice were placed on a metal surface held at a constant temperature (55°C). The time taken to initiate nociceptive behaviors was recorded. A maximum of 20 s was set as cutoff time to prevent tissue damage.

All the mice were allowed to acclimate for 3 days before the hot plate test, and the baseline was measured before drug treatment. Mice were randomly divided into five groups (*n* = 7–8/group); Group 1: vehicle (100 μl, i. p.), Group 2: morphine (10 mg/kg, 100 μl, i. p.), and Group 3–5: IPEA (10, 20, and 40 mg/kg, 100 μl, i. p.), respectively. The hot plate latency was recorded at 30, 60, 90, 120, 180, and 240 min after drug injections. For some experiments, BIC (1 mg/kg, i. p.) or 3-MP (10 mg/kg, i. p.) was injected 30 min before IPEA treatment, and naloxone (1 mg/kg, i. p.) was injected 5 min before IPEA treatment. In the tolerance test, mice were injected with IPEA (20 mg/kg, i. p.) for seven consecutive days, and the hot plate latency was recorded on days 1, 3, 5, and 7. In the case of co-administration of IPEA with morphine to examine the effect on morphine analgesic tolerance, mice were pretreated with intraperitoneal IPEA for 30 min and then administered with 5 mg/kg morphine for eight consecutive days. Subsequent hot plate tests were carried out 30 min after morphine injection on days 1, 3, 5, 7, and 8.

#### CFA-Induced Inflammatory Pain

CFA-induced inflammatory pain is a reliable model of persistent inflammatory hyperalgesia that was initially developed by Larson et al. (Larson et al., 1986). Usually, 20 μl CFA is injected into the plantar surface of the right hind paw to induce inflammation.

Mice were randomly divided into five groups (*n* = 6/group); Group 1: control group, Group 2: CFA group and Group 3–5: IPEA (10, 20, and 40 mg/kg) treatment group. For some experiments, BIC (1 mg/kg, i. p.) was injected 30 min before IPEA treatment. In the acute phase of inflammation, IPEA was administered 4 and 24 h after intraplantar injection of CFA and behavioral parameters were measured at different time points of 1, 2, 3, and 4 h after IPEA injection. In the chronic phase of inflammation, IPEA was administered daily for consecutive 14 days and behavioral parameters were measured 1 h after IPEA treatment on day 3, 7, and 14.

Thermal hyperalgesia was evaluated by using a modified Hargreaves-type hot box as originally described by Hargreaves at al. with some modification (Hargreaves, 1988). In brief, prior to the test the glass surface of hot box was maintained at 30°C and mice were kept in individual boxes for 60 min to habituate the environment. A radiant light source under the glass surface was aligned to the hind paw plantar surface of experimental mice at different time points. A cutoff time of 20 s was set to avoid tissue damage.

Mechanical hyperalgesia was assessed by von Frey filaments (IITC Life Science Inc., CA, United States) as originally described by Chaplan et al. with modification (Chaplan et al., 1994). The calibrated von Frey filament stimulation (ranging from 0.008 to 4 g) was applied to both hind paws. Paw withdrawal thresholds (PTW) were calibrated with a modified up-down method of Dixon (Dixon, 1980). A cutoff pressure of 4 g was set to prevent tissue damage.

#### Rotarod Assay

The rotarod assay was carried out as previously described with some modification (Dunham and Miya, 1957). Mice were trained to remain on the rotarod apparatus before the test session for four trials per day for three consecutive days. The speed was ranging from 5 to 40 rpm and then remained constant. The latency to fall off the rotarod within 300 s were recorded. The hot plate assay was immediately followed and the same groups of mice were used.

#### LPS-Induced Sepsis

Mice were received a single intraperitoneal injection of 10 mg/kg LPS to induce sepsis. IPEA (20 and 40 mg/kg) were treated 1 h prior to LPS injection. Dexamethason (DEX, 5 mg/kg) was used as a positive control. In some experiments, BIC was used and administered (1 mg/kg, i. p.) 30 min before IPEA injection.

At the end of the experimental period, blood and liver samples were collected and stored immediately at −80°C for further analysis.

### Data Analysis

All statistical calculations were performed using GraphPad Prism8.3 (GraphPad, Avenida, CA, United States). Results are expressed as the mean ± SEM. Two-way ANOVA followed by Dunnett post hoc test was used to analyze the data for nociceptive and inflammatory *in vivo* tests; One-way ANOVA followed by Tukey test was used to analyze the data for biochemical tests. *p* < 0.05 was considered significance.

## Results

### The Effect of IPEA in an Acute Pain Model

**FIGURE 1:**
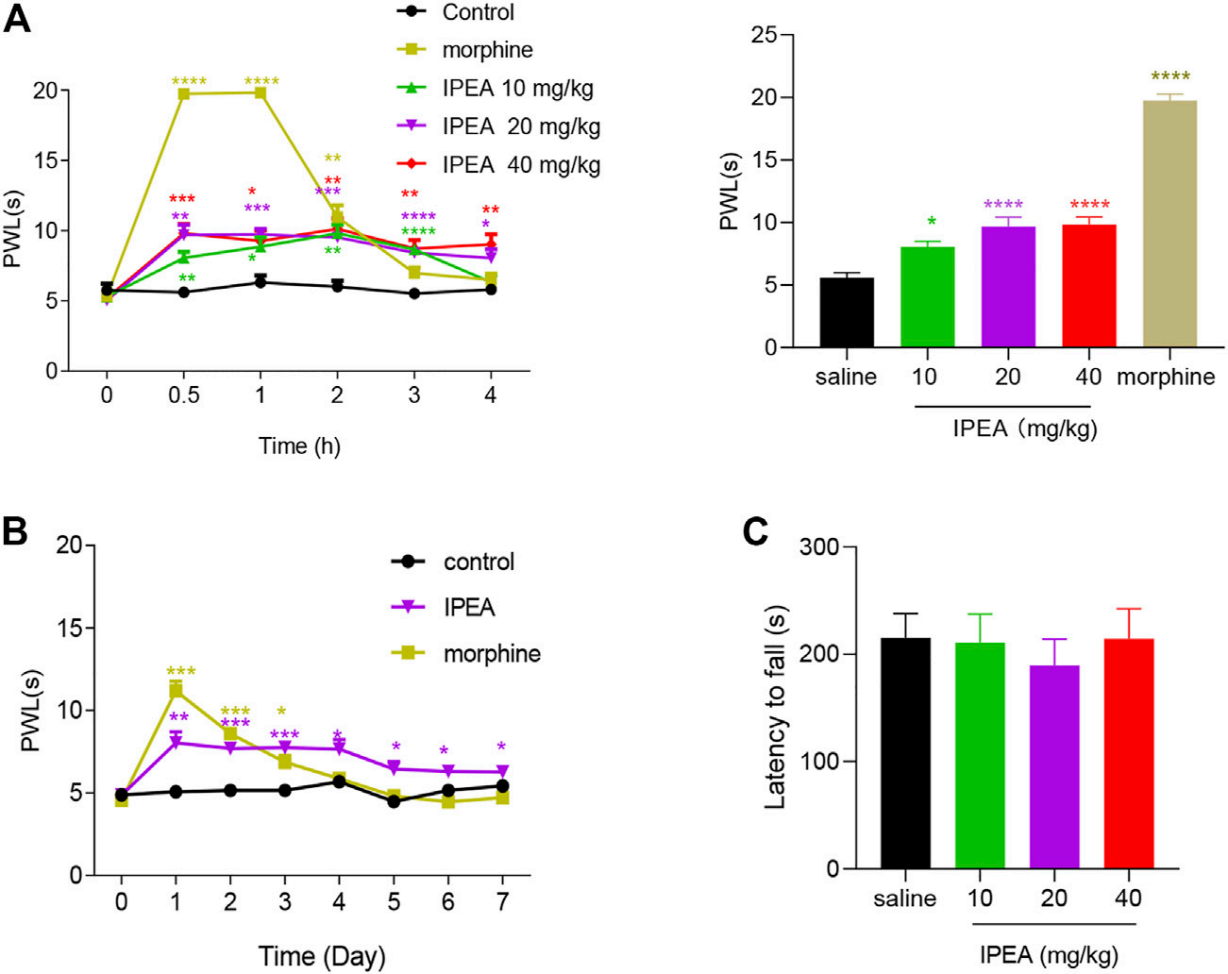
Anti-nociceptive effects of IPEA in acute pain model. **(A)** **Left:** time course of PWL with different doses of IPEA treatment in the hot plate assay. Two-way ANOVA revealed significant drug effects (F4, 31 = 68.02, *p* < 0.0001) and time effects (F4.093,126.9 = 91.44, *p* < 0.0001) followed by Dunnett post hoc test. **Right:** dose-response effect of IPEA on PWL 30 min after administration. One-way ANOVA revealed significant drug effects (F4, 31 = 93.45, *p* < 0.0001) **(B)** Lack of antinociceptive tolerance of IPEA (20 mg/kg). Repeated i. p. injections of 20 mg/kg for seven consecutive days, and PWL was measured 1 h after drugs injection. Morphine was 5 mg/kg. Two-way ANOVA revealed significant drug effects (F2, 14 = 31.02, *p* < 0.0001) and time effect (F4.015,56.02 = 37.69, *p* < 0.0001) followed by Dunnett post hoc test **(C)** Effect of IPEA in rotarod assay. One-way ANOVA was used. Data were presented as the mean ± SEM (*n* = 7-8 mice per group). *p < 0.05, **p < 0.01, ***p < 0.001, ****p < 0.0001 vs Control group.

The acute anti-nociceptive activity of IPEA was measured in the hot plate assay, which recorded responses to a thermal stimulus. As shown in Figure 1A, IPEA was found to induce anti-nociception in a dose-dependent manner. Compared to morphine, IPEA showed a longer lasting anti-nociceptive effect at higher doses (20, 40 mg/kg) for at least 4 h.

The anti-nociceptive tolerance of IPEA was assessed. Mice were subjected to daily administration of IPEA over a 7-day period and were monitored for their responses in the hot plate assay. In contrast to morphine, the effect of IPEA remained effective over the observation period (Figure 1B), indicating that IPEA may lack tolerance.

In order to rule out the possibility that sedation could mask the assessment of pain, IPEA was tested for its potential sedative properties by using rotarod assay. As illustrated in Figure 1C, IPEA did not significantly affect rotarod, suggesting that IPEA did not induce sedation at analgesic doses (10, 20, 40 mg/kg).

### The Effect of IPEA on CFA-Induced Mechanical Allodynia

**FIGURE 2:**
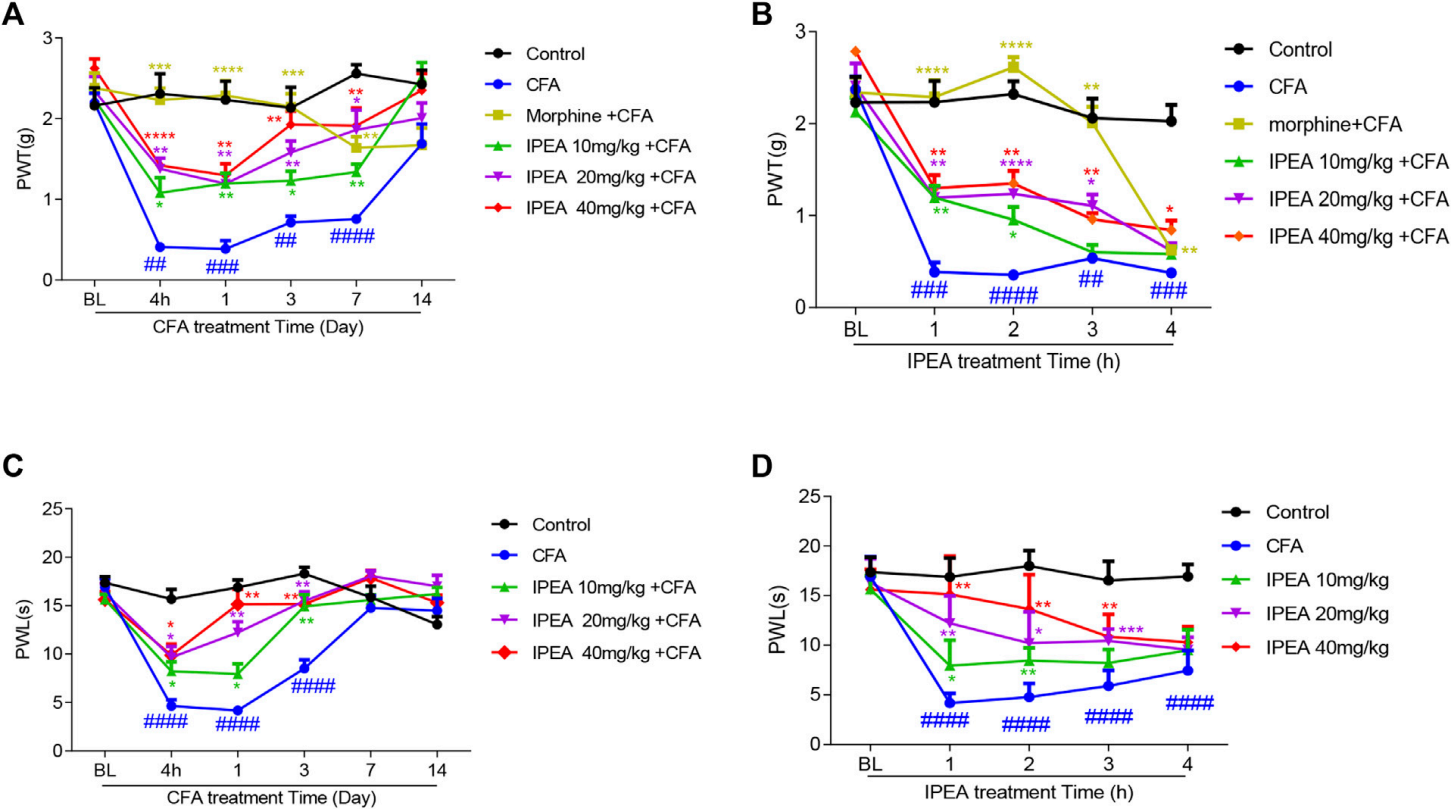
Intraperitoneal injection of IPEA attenuates CFA-induced inflammatory pain of mice. **(A,C)** The time-course of IPEA on CFA-induced chronic inflammatory pain. Mice were daily administered with IPEA (i.p., 10 mg/kg, 20 mg/kg, and 40 mg/kg) after CFA injection. PWT or PWL of IPEA was measured 1 h after IPEA treatment on day 0 (prior to intraplantar injection), 4 h, day 1, day 3, day 7, day 14 after intraplantar injection of CFA. Two-way ANOVA revealed significant drug effects (Figure 2A: F5, 30 = 43.02, *p* < 0.0001; Figure 2C: F4,149 = 30.43) and time effects (Figure 2A: F4.266,128.0 = 28.16, *p* < 0.0001; Figure 2C: F3.781, 112.7 = 43.91, *p* < 0.0001) followed by Dunnett post hoc test **(B and D)** The time-course of IPEA on CFA-induced acute inflammatory pain. Morphine was 5 mg/kg. Two-way ANOVA revealed significant drug effects (Figure 2B: F5, 30 = 40.53, *p* < 0.0001; Figure 2D: F4, 25 = 83.65, *p* < 0.0001) and time effect (Figure 2B: F3.247,97.40 = 118.1, *p* < 0.0001; Figure 2D: F3.150, 78.75 = 43.23, *p* < 0.0001) followed by Dunnett post hoc test. Data were presented as the mean ± SEM (n = 6 mice per group). *p < 0.05, **p < 0.01, ***p < 0.001, ****p < 0.0001 vs CFA group; #*p* < 0.05, ###*p* < 0.001, ####*p* < 0.0001 vs control group.

CFA has been used as a reliable model of persistent inflammatory pain. The mechanical allodynia and thermal hyperalgesia develop approximately 24 h after injection and remain for up to 2 weeks. Prior to implementation of CFA, PWT did not differ between the groups. As shown in Figure 2A, compared with saline control group, the PWT of CFA group was significantly decreased at 4 h, day 1, 3 and 7, indicating that CFA successfully induced mechanical allodynia. The PWT of CFA group rebound on day 14, indicative of the recovery of CFA-induced injury. With daily i. p. injection of IPEA for 14 consecutive days, IPEA significantly increased the PWT at 4 h and days 1, 3, 7, 14 in a dose-dependent manner when compared with the CFA group.

The effect of IPEA was also investigated at different time points on day 1. As shown in Figure 2B the analgesic effect of IPEA was time-dependent and lasted for at least 4 h.

### The Effect of IPEA on CFA-Induced Thermal Hot Hyperalgesia

Intraplantar injection of CFA also resulted in thermal hot hyperalgesia, illustrated by an obvious decrease in the paw withdrawal latency (PWL) during hot plate assay that persisted at least 3 days (Figure 2C). IPEA treatment alleviated CFA-induced thermal hot hyperalgesia from 4 h until day 3 in a dose-dependent manner (Figure 2C).

The effect of IPEA on CFA-induced thermal hot hyperalgesia was also investigated at different time points on day 1. As shown in Figure 2D, the analgesic effect of IPEA was time-dependent and lasted for 3 h.

### Co-administration of IPEA Improved Morphine Analgesic Tolerance

**FIGURE 3:**
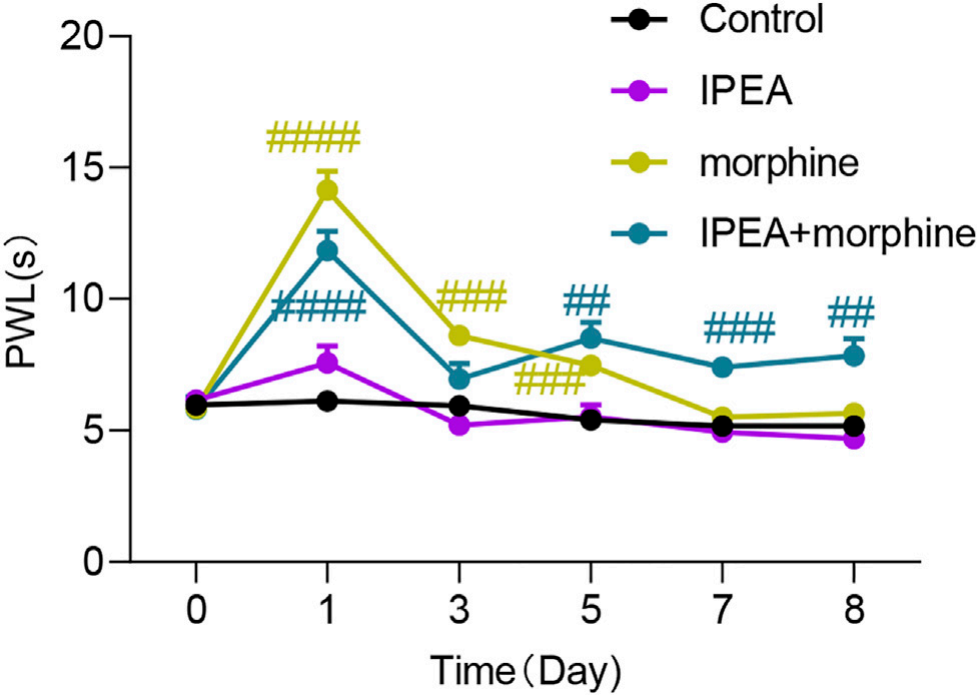
Co-administration of IPEA with morphine improves morphine tolerance. Mice were pretreated with intraperitoneal IPEA (5 mg/kg) for 30 min, followed by 5 mg/kg morphine administration for eight consecutive days. PWL were measured 30 min after morphine injection on day 1, 3, 5, 7 and 8. Two-way ANOVA revealed significant drug effects (F3, 37 = 58.37, *p* < 0.0001) and time effects (F3.362, 123.1 = 53.92, *p* < 0.0001) followed by Dunnett post hoc test. Data were presented as the mean ± SEM (*n* = 8–12 mice per group. #*p* < 0.05, ##*p* < 0.01, ####*p* < 0.0001 vs control.

As shown in Figure 3, the anti-nociception of morphine (5 mg/kg) was observed on day 1 in the hot plate assay. The PWL of morphine was decreased in a time-dependent manner, indicating that tolerance was developed. IPEA at 5 mg/kg did not produce analgesia as shown in Figure 3. However, daily co-administration of 5 mg/kg IPEA with morphine partially restored morphine anti-nociception on day 5, 7, and 8.

### GABA_A_R Antagonist Antagonized the Anti-nociceptive Effect of IPEA

**FIGURE 4:**
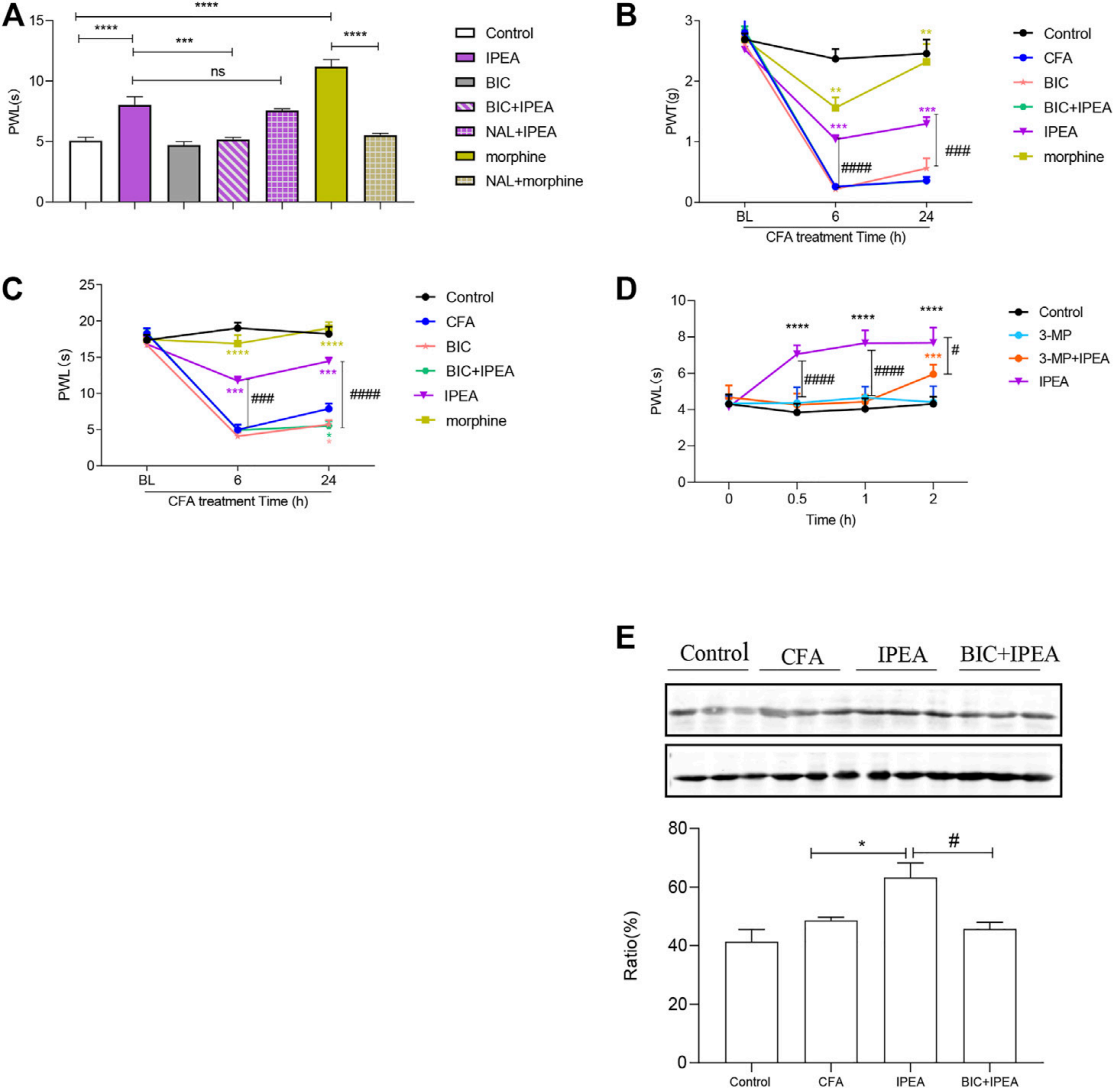
The mechanism of IPEA action in mediating analgesic effects. **(A)** IPEA mediated analgesia through GABA_A_R in an acute pain mouse model. Mice were pretreated with BIC (1 mg/kg, i. p.) for 30 min or naloxone (NAL, 1 mg/kg, opioid receptor antagonist) for 5 min, followed by 20 mg/kg IPEA administration. PWL was measured 1 h after IPEA treatment in the hot plate assay. Unpaired *t* test was used. ***p < 0.001, comparison as indicated. ns, not significant **(B,C)** IPEA mediated analgesia through GABA_A_ receptor in a CFA-induced inflammatory pain mouse model. PWL and PWT were determined 1 h after IPEA treatment. Mice were pre-treated with BIC (1 mg/kg, i. p.) for 30 min, followed by 20 mg/kg IPEA administration. Two-way ANOVA revealed significant drug effects (**(B)**: F5, 30 = 39.42, *p* < 0.0001; **(C)**: F5, 30 = 97.55, *p* < 0.0001) and time effects (**(B)**: F1.692, 50.77 = 323.6; **(C)**: F1.889, 56.66 = 153.0, *p* < 0.0001) followed by Dunnett post hoc test. **p < 0.01, ***p < 0.001, ****p < 0.0001 vs CFA group; #*p* < 0.05, ###*p* < 0.001, ####*p* < 0.0001, comparison as indicated. **(D)** Mice were pretreated with 3-MP (10 mg/kg) for 30 min, followed by 20 mg/kg IPEA. PWL of mice was measured at different time points in the hot plate assay. Two-way ANOVA revealed significant drug effects (F3, 28 = 64.01, *p* < 0.0001) and time effects (F2.568, 71.90 = 17.07, *p* < 0.0001) followed by Dunnett post hoc test. ***p < 0.001, ****p < 0.0001 vs saline control group; #*p* < 0.05, ####*p* < 0.0001, comparison as indicated. **(E)** The expression levels of GABA_A_Rα2 in the spinal cord of CFA-induced inflammatory pain mice. Mice were pretreated with IPEA (20 mg/kg) for 1 h and the spinal cord was collected 24 h after CFA treatment. Unpaired *t* test was used. The expression levels of GABA_A_Rα2 in the spinal cord were determined by western blot. *p < 0.05, #*p* < 0.05, comparison as indicated. Data were obtained from three independent experiments and expressed as the means ± SEM (*n* = 6–8 mice per group).

IPEA (20 mg/kg) increased the hot plate latency in the acute pain model as seen in Figure 4A. However, BIC, a selective GABA_A_R antagonist significantly reversed IPEA anti-nociception. In contrast, naloxone, a selective opioid receptor antagonist, was unable to affect the effect of IPEA (Figure 4A).

In CFA-induced inflammatory pain model, BIC completely blocked the anti-nociceptive effect of IPEA at 6 h, and partially inhibited at 24 h after CFA injection (Figures 4B,C).

### Glutamate Decarboxylase Inhibitor Reversed the Anti-nociceptive Effect of IPEA

GABA is one of the inhibitory neurotransmitters and formed by glutamate *via* glutamate decarboxylase (GAD). In order to determine the role of endogenous GABA in the anti-nociceptive effect of IPEA, 3-MP, a GAD inhibitor, was used in the hot plate assay. As illustrated in Figure 4D, 3-MP significantly blocked the anti-nociceptive effects of IPEA at different time points. We therefore, inferred that the anti-nociceptive effect of IPEA might be mediated, at least in part, by the endogenous inhibitory neurotransmitter GABA in the present study.

### IPEA Increased the Expression of GABA_A_Rα2

The expression of GABA_A_Rα2 was measured by immunoblotting. As shown in Figure 4E, the protein expression level of GABA_A_Rα2 was unaffected in CFA-induced inflammatory pain model. However, IPEA (20 mg/kg) treatment mice showed a significantly increase in GABA_A_Rα2 expression. In addition, BIC was able to reverse IPEA-induced GABA_A_Rα2 expression.

### IPEA Exerted Anti-Inflammatory Effect *via* GABA_A_R in LPS Stimulated RAW 264.7 Macrophage Cells

**FIGURE 5:**
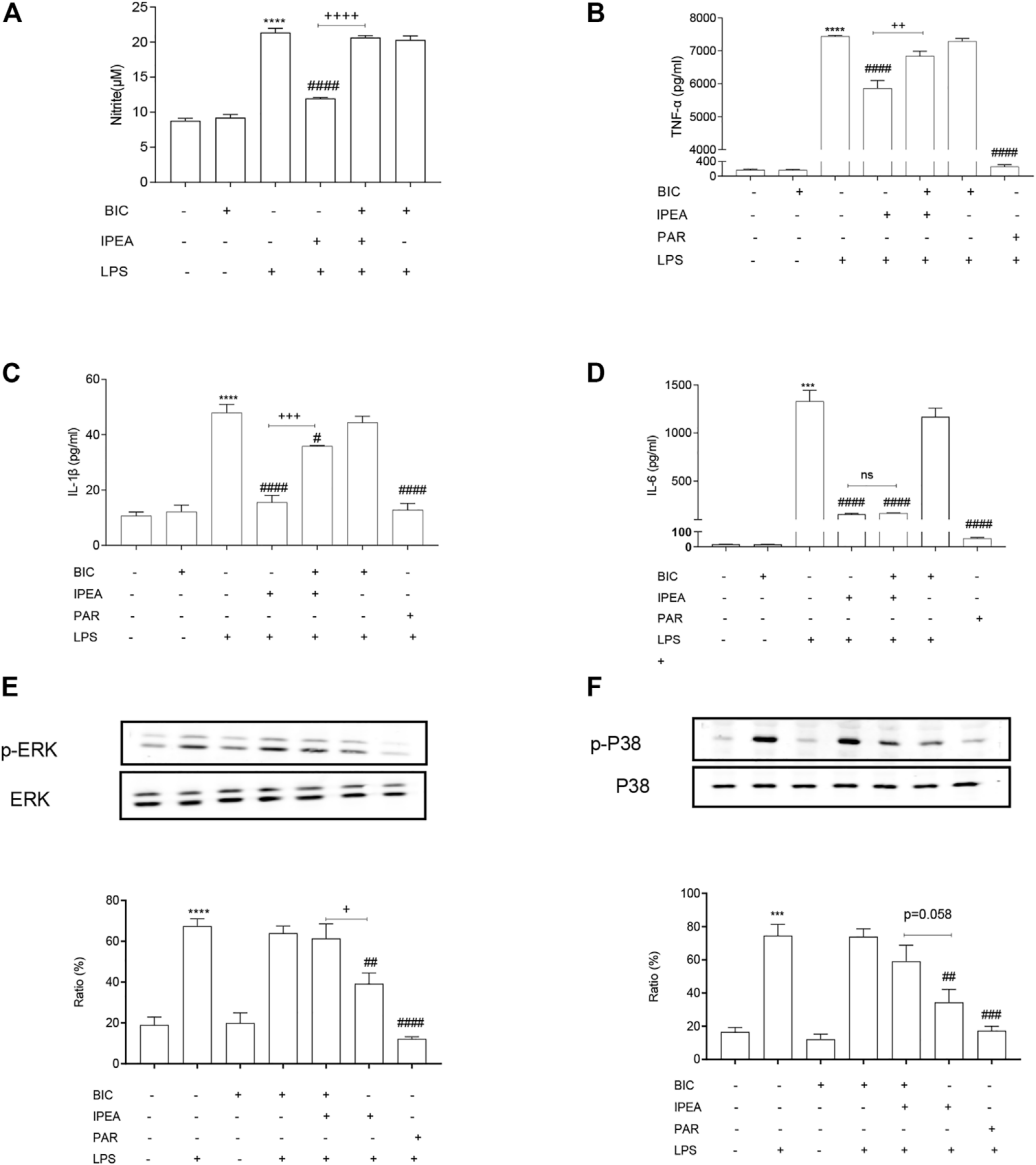
IPEA exerts anti-inflammatory effect via GABA_A_R in LPS stimulated RAW 264.7 macrophage cells. **(A)** Effects of BIC on IPEA-induced suppression on NO production. Cells were pretreated with BIC (1 μM) for 10 min, followed by 1-h IPEA (10 μg/ml) treatment and then stimulated with LPS (100 ng/ml) for 23 h. The nitrite level was determined by Griess reagent. **(B–D)** Effects of BIC on IPEA-induced suppression on the expression levels of inflammatory cytokines. The protein expression levels of TNF-α **(B)**, IL-1β **(C)** and IL-6 **(D)** were determined by ELISA according to manufacturer’s instructions. Cells were pretreated with BIC (1 μM) for 10 min, followed by 1-h IPEA (10 μg/ml) treatment, and then stimulated with LPS (100 ng/ml) for 4 h. **(E,F)** Effects of BIC on IPEA-induced suppression on signaling pathways of MAPKs. Phosphorylated ERK (*p*-ERK) **(E)**, and P38 (p-P38) **(F)**, were determined by Western blot and normalized to the total ERK and P38 expression levels, respectively. Cells were pretreated with BIC (1 μM) for 10 min, followed by IPEA (10 μg/ml) treatment for 1 h, and then stimulated with LPS (100 ng/ml) for 30 min. PAR (parthenolide, 10 μM) was used as positive control. One-way ANOVA followed Tukey test was used for data analysis. The data were obtained from three independent experiments and expressed as the means ± SEM. *p < 0.05, **p < 0.01, ***p < 0.001, ****p < 0.0001 vs the control group; ##*p* < 0.01, ###*p* < 0.001, ####*p* < 0.0001 vs LPS-simulated group; +*p* < 0.05, ++*p* < 0.01, +++*p* < 0.001, compared between IPEA group and BIC + IPEA group; ns, not significant.

As shown in Figures 5A–D, BIC significantly attenuated IPEA-induced suppression of NO production and expression levels of TNF-α and IL-1β, but not IL-6 (Figure 5D), in LPS stimulated RAW 264.7 macrophage cells. BIC also reversed IPEA-induced inhibition of ERK/P38 MAPKs signaling pathway (Figures 5E,F).

### IPEA Exerts Anti-Inflammatory Effect *via* GABA_A_R in the Mouse Model of LPS-Induced Sepsis

**FIGURE 6:**
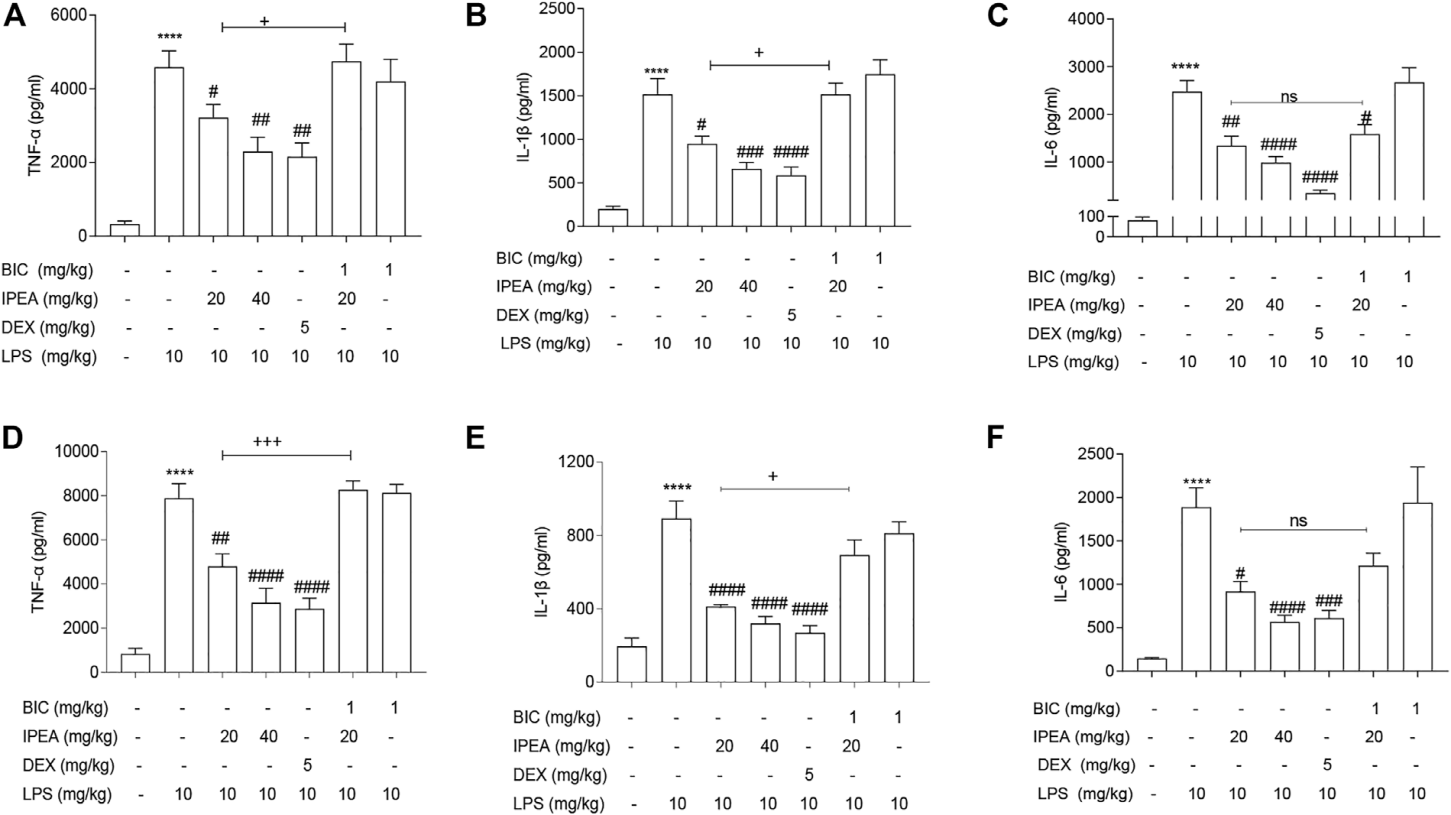
IPEA exerts anti-inflammatory effect via GABAAR in septic mice. The protein expression levels of TNF-α, IL-1β and IL-6 in serum **(A–C)**, and in the liver **(D–F)** were determined by ELISA according to manufacturer’s instructions. Mice were pretreated with BIC (1 mg/kg) for 30 min, followed by IPEA administration (20 and 40 mg/kg, i. p.). After 1 h-IPEA treatment, mice were injected with LPS (10 mg/kg, i. p.). After 12 h, mice liver tissue was obtained, and the serum was collected by centrifuging at 4°C, 4,000 rpm for 10 min. The liver tissue was treated with animal tissue homogenizer, and then centrifuged at 4°C, 1,000 rpm for 20 min. The supernatant was obtained and quantitated by BCA kit. DEX (dexamethason, 5 mg/kg) was used as positive control. One-way ANOVA followed Tukey test was used for data analysis. The data were expressed as the means ± SEM (n = 6–7). *p < 0.05, **p < 0.01, ***p < 0.001, ****p < 0.0001 vs the control group; #*p* < 0.05, ##*p* < 0.01, ###*p* < 0.001, ####*p* < 0.0001 vs LPS treatment group; +*p* < 0.05, ++*p* < 0.01, +++*p* < 0.001, compared between IPEA group and BIC + IPEA group. ns, not significant.

The mouse model of sepsis was established to explore the anti-inflammatory effect of IPEA *in vivo*. As shown in Figures 6A–F, the expression of pro-inflammatory mediators TNF-α, IL-6, and IL-1β in serum and liver tissues of LPS-induced septic mice were significantly elevated when compared with the control group. Treatment with 20 and 40 mg/kg IPEA or DEX significantly reduced the levels of these inflammatory mediators.

BIC could reverse all these effects (Figures 6A,B and Figures 6D,E), except IL-6 (Figures 6C,F), which was consistent with the *in vitro* data (Figure 5D).

## Discussion

Pain is an uncomfortable sensory and emotional experience associated with tissue lesions. Pain has become the third common health issue that affects more individuals than heart disease and cancer (Loeser and Melzack, 1999). As part of our ongoing project of discovering new analgesics, the present study aims to investigate the analgesic properties of *I. pterocaula*, a folk medicine in China.

Our results revealed that IPEA markedly and long-lastingly reversed thermally induced acute pain (i e., the hot plate assay). Mechanism study revealed that both GABA_A_R antagonist-BIC, and GAD inhibitor-3-MP, reversed IPEA-induced decrease of PTW in the hot plate assay, suggesting the contribution of GABA/GABA_A_R system in the acute anti-nociceptive effect of IPEA. IPEA also significantly alleviated the CFA induced persistent pain. The expression level of GABA_A_Rα2 was increased with IPEA treatment in CFA-induced pain model, providing further evidence of functional role of GABA/GABA_A_R in mediating the analgesic effect of IPEA. The subtype-selective benzodazepine-site ligands have been reported to be highly effective against inflammatory and neuropathic pain yet devoid of unwanted sedation and tolerance development (Knabl et al., 2008; Knabl et al., 2009; Munro et al., 2009; Diet al., 2011). Therefore, it is possible that IPEA may selectively activate GABA_A_R (e.g., GABA_A_Rα2) to produce analgesia but without sedation. Considerable evidence supported that facilitating GABAergic inhibition was able to reverse pathological pain states that resulted from reduced presynaptic GABA release or from reduced responsiveness of postsynaptic GABA_A_Rs (Moore et al., 2002; Kami et al., 2016). We speculate that IPEA produced analgesic effects, at least in part, by increasing the synthesis of GABA through activation of GABA-synthesizing enzyme GAD. GABA was the endogenous GABA_A_R, so increased GABA could markedly induce GABA_A_R activity. However, since IPEA is a multi-components fraction, it cannot be excluded that some compounds in IPEA may bind and activate GABA_A_R directly. The detailed mechanism remains to be further studied.

The behavior of IPEA was also tested against anti-nociceptive tolerance. IPEA didn’t develop tolerance with daily administration for 7 days. In addition, IPEA at a non-antinoceptive dose showed the ability to improve morphine tolerance. Therefore, IPEA may have an advantage over morphine in treating chronic pain or can be combined with morphine clinically. These results also suggested a possible interaction of GABA-opioids in addition of a direct GABA action.

In our previous work, IPEA showed anti-inflammatory effect in LPS-induced RAW 264.7 macrophages, which prompted us two questions: 1) whether IPEA possesses anti-inflammatory property *in vivo*, and 2) whether GABA_A_R also mediates the anti-inflammatory effect of IPEA. Therefore, in this study, we evaluated the potential anti-inflammatory effect of IPEA in LPS-induced sepsis of mice, and further explored whether its underlying mechanism involved GABA_A_R. Our results showed that IPEA significantly suppressed the expression levels of pro-inflammatory cytokines, including IL-1β, IL-6, and TNF-α in both the serum and lung tissue of the septic mouse models. BIC could reverse the anti-inflammatory action of IPEA in both LPS-induced RAW 264.7 macrophages and the mouse model of sepsis, suggesting that IPEA also exerted its anti-inflammation *via* GABA_A_R. Some recent studies have shown that non-neuronal cell types also produced GABA outside the CNS, particularly immune cells (Prud'Homme et al., 2015). GABA has been reported to suppress immune-mediated pro-inflammatory responses by its specific receptors expressed on immune cells (Prud’homme et al.; Han et al., 2007; Wang et al., 2012; Yocum et al., 2017; Ngo and Vo, 2019). GABA has also been used in the treatment of some immune-related diseases, such as rheumatoid arthritis (Tian et al., 2004; Reyes-Garcia et al., 2007; Kelley et al., 2008). In our study, BIC reversed IPEA-induced suppressing signaling pathways and the production of pro-inflammatory cytokines, indicating that GABA/GABA_A_R neurotransmission system participated the anti-inflammatory activity of IPEA. We propose that IPEA might increase the levels of endogenous GABA, which subsequently binds to GABA_A_R and downregulates ERK and p38 MAPK activity, thus reducing peripheral production of pro-inflammatory cytokines. This speculation is consistent with the published report in which the expression of splenic IL-1β and TNF-α were attenuated by the administration of GABA or its analogues (Crowley et al., 2016). However, the expression level of IL-6 was unaffected by BIC, further supporting the possibility of multiple targets of IPEA. In addition, suppression of the pro-inflammatory mediators might also contribute to the anti-nociceptive effect of IPEA observed in CFA induced pain models since these pro-inflammatory mediators could modulate the nociceptors (Oh et al., 2001; Basbaum et al., 2009; Gold and Gebhart, 2010).

Our data from the pain and inflammation models show that GABA_A_R plays a key role in the pharmacological effects of IPEA. *I. pterocaula* may be useful as a complementary or alternative therapeutic agent for the treatment of pain and inflammation. Further studies will be conducted to isolate, characterize and elucidate the active ingredients of IPEA responsible for the observed pharmacological effects.

## Data Availability

The raw data supporting the conclusions of this article will be made available by the authors, without undue reservation.

## Abbreviations

BIC, (+)-bicuculline; CFA, Complete Freund’s adjuvant; EA, ethyl acetate; GABAAR, GABAA receptor; GABA, γ-aminobutyric acid; GAD, glutamate decarboxylase; I. pterocaula, Inula pterocaula Franch; IPEA, the EA fraction of Inula pterocaula Franch; LPS, lipopolysaccharide; 3-MP, 3-mercaptopropanoic acid; NO, nitric oxide; PE, petroleum ether; PWL, paw withdrawal latency; PWT, paw withdrawal threshold.

## References

[B1] Amin S. Kaloo Z. A. Singh S. Altaf T. (2013). Medicinal Importance of Genus Inula- a Review. Ijcrr 5, 170–177. 10.5897/JPP2013.0285

[B2] Basbaum A. I. Bautista D. M. Scherrer G. Julius D. (2009). Cellular and Molecular Mechanisms of Pain. Cell 139, 267–284. 10.1016/j.cell.2009.09.028 19837031 PMC2852643

[B3] Bhat R. Axtell R. Mitra A. Miranda M. Lock C. Tsien R. W. (2010). Inhibitory Role for GABA in Autoimmune Inflammation. Proc. Natl. Acad. Sci. U S A. 107, 2580–2585. 10.1073/pnas.0915139107 20133656 PMC2823917

[B4] Bowsher D., (1993). Paradoxical Pain. BMJ 306, 473–474. 10.1136/bmj.306.6876.473 8448453 PMC1676821

[B5] Chaplan S. R. Bach F. W. Pogrel J. W. Chung J. M. Yaksh T. L. (1994). Quantitative Assessment of Tactile Allodynia in the Rat Paw. J. Neurosci. Methods 53, 55–63. 10.1016/0165-0270(94)90144-9 7990513

[B6] Committee, E. (1979). Flora of China. Science Press. (Beijing) & Missouri Botanical Garden (St. Louis).

[B7] Crowley T. Cryan J. F. Downer E. J. O'leary O. F. (2016). Inhibiting Neuroinflammation: The Role and Therapeutic Potential of GABA in Neuro-Immune Interactions. Brain Behav. Immun. 54, 260–277. 10.1016/j.bbi.2016.02.001 26851553

[B8] Dawes J. M. Calvo M. Perkins J. R. Paterson K. J. Kiesewetter H. Hobbs C. (2011). CXCL5 Mediates UVB Irradiation-Induced Pain. Sci. Transl. Med. 3, 90ra60. 10.1126/scitranslmed.3002193 PMC3232447 21734176

[B9] Derry S. Moore R. A. Gaskell H. Mcintyre M. Wiffen P. J. (2015).Topical NSAIDs for Acute Musculoskeletal Pain in Adults. Cochrane Database Syst. Rev. 2015, CD007402. 10.1002/14651858.CD007402 PMC6426435 26068955

[B10] Di Lio A. Benke D. Besson M. Desmeules J. Daali Y. Wang Z. J. (2011). HZ166, a Novel GABAA Receptor Subtype-Selective Benzodiazepine Site Ligand, Is Antihyperalgesic in Mouse Models of Inflammatory and Neuropathic Pain. Neuropharmacology 60, 626–632. 10.1016/j.neuropharm.2010.11.026 21145329 PMC3566476

[B11] Dixon W. J. (1980). Efficient Analysis of Experimental Observations. Annu. Rev. Pharmacol. Toxicol. 20, 441–462. 10.1146/annurev.pa.20.040180.002301 7387124

[B12] Dodrill C. L. Helmer D. A. Kosten T. R. (2011). Prescription Pain Medication Dependence. Am. J. Psychiatry 168, 466–471. 10.1176/appi.ajp.2010.10020260 21536702

[B13] Dunham N. W. Miya T. S. (1957). A Note on a Simple Apparatus for Detecting Neurological Deficit in Rats and Mice. J. Am. Pharm. Assoc. Am. Pharm. Assoc. 46, 208–209. 10.1002/jps.3030460322 13502156

[B14] Farrant M. Nusser Z. (2005). Variations on an Inhibitory Theme: Phasic and Tonic Activation of GABA(A) Receptors. Nat. Rev. Neurosci. 6, 215–229. 10.1038/nrn1625 15738957

[B15] Fischer B. D. (2017). GABAA Receptors as Targets for the Management of Pain-Related Disorders: Historical Perspective and Update. CNS. Neurol. Disord. Drug Targets 16, 658–663. 10.2174/1871527316666170207155149 28176641

[B16] Ge M. M. Chen S. P. Zhou Y. Q. Li Z. Tian X. B. Gao F. (2019). The Therapeutic Potential of GABA in Neuron-Glia Interactions of Cancer-Induced Bone Pain. Eur. J. Pharmacol. 858, 172475. 10.1016/j.ejphar.2019.172475 31228456

[B17] Gold M. S. Gebhart G. F. (2010). Nociceptor Sensitization in Pain Pathogenesis. Nat. Med. 16, 1248–1257. 10.1038/nm.2235 20948530 PMC5022111

[B18] Han D. Kim H. Y. Lee H. J. Shim I. Hahm D. H. (2007). Wound Healing Activity of Gamma-Aminobutyric Acid (GABA) in Rats. J. Microbiol. Biotechnol. 17, 1661–1669. 10.1016/j.mimet.2007.08.002 18156782

[B19] Hargreaves K. Dubner R. Brown F. Flores C. Joris J. (1988). A New and Sensitive Method for Measuring thermal Nociception in Cutaneous Hyperalgesia. Pain 32 (1), 77–88. 10.1016/0304-3959(88)90026-7 3340425

[B20] Huang C. L. Wu L. H. Yu Y. Jin H. Z. Zhang Y. (2021). The Anti-inflammatory Activity and Mechanisms of Inula Pterocaula Extrtct in LPS-Induced Inflammatory Responses in RAW 264.7 Macrophages. J. Dalian polytechnic Univ. 40, 253–259.

[B21] Ji R. R. Nackley A. Huh Y. Terrando N. Maixner W. (2018). Neuroinflammation and Central Sensitization in Chronic and Widespread Pain. Anesthesiology 129, 343–366. 10.1097/ALN.0000000000002130 29462012 PMC6051899

[B22] Jia M. Li X. (2005). ZHONGGUO MINZU YAOZHIYAO China: The Medicine Science and Technology Press of China. Beijing, China: China Medical Science Press.

[B23] Kami K. Taguchi Ms S. Tajima F. Senba E. (2016). Improvements in Impaired GABA and GAD65/67 Production in the Spinal Dorsal Horn Contribute to Exercise-Induced Hypoalgesia in a Mouse Model of Neuropathic Pain. Mol. Pain 12, 1744806916629059. 10.1177/1744806916629059 27030712 PMC4956002

[B24] Kelley J. M. Hughes L. B. Bridges S. L. Jr. (2008). Does Gamma-Aminobutyric Acid (GABA) Influence the Development of Chronic Inflammation in Rheumatoid Arthritis? J. Neuroinflammation 5, 1. 10.1186/1742-2094-5-1 18171484 PMC2235846

[B25] Knabl J. Witschi R. Hösl K. Reinold H. Zeilhofer U. B. Ahmadi S. (2008). Reversal of Pathological Pain through Specific Spinal GABAA Receptor Subtypes. Nature 451, 330–334. 10.1038/nature06493 18202657

[B26] Knabl J. Zeilhofer U. B. Crestani F. Rudolph U. Zeilhofer H. U. (2009). Genuine Antihyperalgesia by Systemic Diazepam Revealed by Experiments in GABAA Receptor point-mutated Mice. Pain 141, 233–238. 10.1016/j.pain.2008.10.015 19091469

[B27] Larson A. A. Brown D. R. El-Atrash S. Walser M. M. (1986). Pain Threshold Changes in Adjuvant-Induced Inflammation: a Possible Model of Chronic Pain in the Mouse. Pharmacol. Biochem. Behav. 24, 49–53. 10.1016/0091-3057(86)90043-2 3945666

[B28] Loeser J. D. Melzack R. (1999). Pain: an Overview. Lancet 353, 1607–1609. 10.1016/S0140-6736(99)01311-2 10334273

[B29] Long Z. Zhang Y. Guo Z. Wang L. Xue X. Zhang X. (2014). Amide Alkaloids from Scopolia Tangutica. Planta. Med. 80, 1124–1130. 10.1055/s-0034-1382961 25127021

[B30] Lüddens H. Korpi E. R. (1995). Biological Function of GABAA/benzodiazepine Receptor Heterogeneity. J. Psychiatr. Res. 29, 77–94. 10.1016/0022-3956(94)00040-x 7545236

[B31] Moore K. A. Kohno T. Karchewski L. A. Scholz J. Baba H. Woolf C. J. (2002). Partial Peripheral Nerve Injury Promotes a Selective Loss of GABAergic Inhibition in the Superficial Dorsal Horn of the Spinal Cord. J. Neurosci. 22, 6724–6731. 12151551 10.1523/JNEUROSCI.22-15-06724.2002 PMC6758148

[B32] Munro G. Ahring P. K. Mirza N. R. (2009). Developing Analgesics by Enhancing Spinal Inhibition after Injury: GABAA Receptor Subtypes as Novel Targets. Trends. Pharmacol. Sci. 30, 453–459. 10.1016/j.tips.2009.06.004 19729210

[B33] Neumann E. Küpfer L. Zeilhofer H. U. (2021). The α2/α3GABAA Receptor Modulator TPA023B Alleviates Not Only the Sensory but Also the Tonic Affective Component of Chronic Pain in Mice. Pain 162, 421–431. 10.1097/j.pain.0000000000002030 32773599 PMC7808355

[B34] Ngo D. H. Vo T. S. (2019). An Updated Review on Pharmaceutical Properties of Gamma-Aminobutyric Acid. Molecules 24. 10.3390/molecules24152678 PMC6696076 31344785

[B35] Oh S. B. Tran P. B. Gillard S. E. Hurley R. W. Hammond D. L. Miller R. J. (2001). Chemokines and Glycoprotein120 Produce Pain Hypersensitivity by Directly Exciting Primary Nociceptive Neurons. J. Neurosci. 21, 5027–5035. 10.1523/jneurosci.21-14-05027.2001 11438578 PMC6762869

[B36] Olsen R. W. (2018). GABAA Receptor: Positive and Negative Allosteric Modulators. Neuropharmacology 136, 10–22. 10.1016/j.neuropharm.2018.01.036 29407219 PMC6027637

[B37] Prud'homme G. J. Glinka Y. Wang Q. (2015). Immunological GABAergic Interactions and Therapeutic Applications in Autoimmune Diseases. Autoimmun. Rev. 14, 1048–1056. 10.1016/j.autrev.2015.07.011 26226414

[B38] Reyes-García M. G. Hernández-Hernández F. Hernández-Téllez B. García-Tamayo F. (2007). GABA (A) Receptor Subunits RNA Expression in Mice Peritoneal Macrophages Modulate Their IL-6/IL-12 Production. J. Neuroimmunol. 188, 64–68. 10.1016/j.jneuroim.2007.05.013 17599468

[B39] Rudolph U. Knoflach F. (2011). Beyond Classical Benzodiazepines: Novel Therapeutic Potential of GABAA Receptor Subtypes. Nat. Rev. Drug Discov. 10, 685–697. 10.1038/nrd3502 21799515 PMC3375401

[B40] Seifi M. Rodaway S. Rudolph U. Swinny J. D. (2018). GABAA Receptor Subtypes Regulate Stress-Induced Colon Inflammation in Mice. Gastroenterology 155, 852–e3. 10.1053/j.gastro.2018.05.033 29802853

[B41] Tian J. Lu Y. Zhang H. Chau C. H. Dang H. N. Kaufman D. L. (2004). Gamma-aminobutyric Acid Inhibits T Cell Autoimmunity and the Development of Inflammatory Responses in a Mouse Type 1 Diabetes Model. J. Immunol. 173, 5298–5304. 10.4049/jimmunol.173.8.5298 15470076

[B42] Wang D. S. Zurek A. A. Lecker I. Yu J. Abramian A. M. Avramescu S. (2012). Memory Deficits Induced by Inflammation Are Regulated by α5-subunit-containing GABAA Receptors. Cell Rep 2, 488–496. 10.1016/j.celrep.2012.08.022 22999935 PMC4391624

[B43] Wang L. Zhang Y. Wang Z. Gong N. Kweon T. D. Vo B. (2016). The Antinociceptive Properties of the Corydalis Yanhusuo Extract. PLoS One 11, e0162875. 10.1371/journal.pone.0162875 27622550 PMC5021270

[B44] Woolfe G. Macdonald A. D. (1944). THE EVALUATION OF THE ANALGESIC ACTION OF PETHIDINE HYDROCHLORIDE (DEMEROL). J. Pharmacol. Exp. Ther. 80, 300–307.

[B45] Wu L. Fan Y. Fan C. Yu Y. Sun L. Jin Y. (2017). Licocoumarone Isolated from Glycyrrhiza Uralensis Selectively Alters LPS-Induced Inflammatory Responses in RAW 264.7 Macrophages. Eur. J. Pharmacol. 801, 46–53. 10.1016/j.ejphar.2017.02.049 28263754

[B46] Yocum G. T. Turner D. L. Danielsson J. Barajas M. B. Zhang Y. Xu D. (2017). GABAA Receptor α4-subunit Knockout Enhances Lung Inflammation and Airway Reactivity in a Murine Asthma Model. Am. J. Physiol. Lung Cel Mol. Physiol. 313, L406–L415. 10.1152/ajplung.00107.2017 PMC5582940 28473323

[B47] Zhang Y. Wang C. Guo Z. Zhang X. Wang Z. Liang X. (2014). Discovery of N-Methyltetrahydroprotoberberines with κ-opioid Receptor Agonists-Opioid Receptor Agonist Activities from Corydalis Yanhusuo W. T. Wang by Using Two-Dimensional Liquid Chromatography. J. Ethnopharmacol. 155, 1597–1602. 10.1016/j.jep.2014.07.057 25107388

[B48] Zhang Y. Wang C. Wang L. Parks G. S. Zhang X. Guo Z. (2014). A Novel Analgesic Isolated from a Traditional Chinese Medicine. Curr. Biol. 24, 117–123. 10.1016/j.cub.2013.11.039 24388848 PMC3912990

[B49] Zhu S. Noviello C. M. Teng J. Walsh R. M. Kim J. J. Hibbs R. E. (2018). Structure of a Human Synaptic GABAA Receptor. Nature 559, 67–72. 10.1038/s41586-018-0255-3 29950725 PMC6220708

[B50] Zhu S. L. Huang C. L. Xiao Y. Z. Xie Y. G. Jin H. Z. (2019). Pterocaullins A-D, Four Sesquiterpene Lactones from Inula Pterocaula. Phytochemistry Letters. 33, 70–76. 10.1016/j.phytol.2019.07.010

